# Levels of shared autonomy in brain-robot interfaces: enabling multi-robot multi-human collaboration for activities of daily living

**DOI:** 10.3389/fnhum.2025.1718713

**Published:** 2026-01-15

**Authors:** Hannah Douglas, Marina Di Vincenzo, Rousslan Fernand Julien Dossa, Luca Nunziante, Shivakanth Sujit, Kai Arulkumaran

**Affiliations:** Araya Inc., Tokyo, Japan

**Keywords:** brain-robot interface, collaborative robot, human-robot interaction, imitation learning, mobile manipulation, multi-agent, shared autonomy

## Abstract

Individuals with ALS and other severe motor impairments often rely on caregivers for daily tasks, which limits their independence and sense of control. Brain-robot interfaces (BRIs) have the potential to restore autonomy, but many existing systems are task-specific and highly automated, which reduces the users' sense of empowerment and limits opportunities to exercise autonomy. In particular, shared autonomy approaches hold promise for overcoming current BRI limitations, by balancing user control with increased robot capabilities. In this work, we introduce a collaborative BRI that integrates non-invasive EEG, EMG, and eye tracking to enable multi-user, multi-robot interaction in a shared kitchen environment with mobile manipulators. Our system modulates assistance through three levels of autonomy—Assisted Teleoperation, Shared Autonomy, and Full Automation—allowing users to retain meaningful control over task execution while reducing effort for routine operations. We conducted a controlled user study comparing autonomy conditions, evaluating performance, workload, ease of use, and agency. Our results show that, while Full Automation was generally preferred by users due to lower workload and higher usability, Shared Autonomy provided higher reliability and preserved user agency, especially in the presence of noisy EEG decoding. Although there was significant individual variability in EEG decoding performance, our *post-hoc* analysis revealed the potential benefits of customizing pipelines for each user. Finally, we note that our findings are specific to the multi-modal configuration tested and should not be interpreted as a universal claim about the superiority of any autonomy level, and, furthermore, our user study was limited by the use of healthy adults rather than target population (e.g., individuals with ALS), gender imbalance, and a relatively small sample size, which may affect generalizability. Project website: https://coopopen.github.io/.

## 1 Introduction

In the present day, around 500,000 people suffer from amyotrophic lateral sclerosis (ALS) or similar mobility limitations. These physical limitations often prevent users from performing various activities of daily living (ADLs), requiring these individuals to resort to hiring care-takers for support. While caregiving services are invaluable, they can impose a significant financial burden on both individuals and their families, and have also been associated with psychological effects such as feelings of isolation and helplessness for the user (Cree et al., 2020).

Beyond individual assistance, there is growing recognition of the importance of creating collaborative spaces that empower individuals with mobility impairments. However, these collaborative environments remain limited in number, highlighting the need for assistive technologies that can support multi-user cooperation and shared task execution. Dawn Cafe represents one of the few examples, where technology creates intentional spaces for individuals with disabilities to socialize while giving them a sense of agency in spaces where they typically are not included (Barbareschi et al., 2023). However, even within existing spaces such as Dawn Cafe, the physical capabilities of the robots are typically limited.

To address these physical limitations, physically assistive robots (PARs) offer a promising avenue by helping individuals complete ADLs such as housework (Cabrera et al., 2021), eating (Al-Halimi and Moussa, 2016), and grooming (Hawkins et al., 2014). While the design of these PARs differs greatly across systems, the majority are optimized for a single, well-defined task (e.g., grasping a cup or moving a cursor), and struggle to generalize across the diverse range of activities of daily living required for real-world independence (Nanavati et al., 2023). In contrast, there is considerable interest in generalist, multi-task robot controllers in the broader robotics research community (Brohan et al., 2023; Zitkovich et al., 2023; Ghosh et al., 2024; Black et al., 2024)—a trend that we echo in our proposed system.

Interfacing with PARs remains another major barrier for those with severe impairments. For individuals with severe mobility impairments, such as those in advanced stages of ALS, interfacing with and controlling these robots via methods such as joysticks, touchscreens or voice commands poses a significant challenge due to the limited motor capabilities. Brain-robot interfaces (BRIs) have emerged as a possible solution to this problem.^1^ In recent years, non-invasive BRIs have gained much attention, allowing for individuals to control external devices without physical movement or surgical intervention. These systems typically rely on electroencephalography (EEG) or other external sensing modalities to detect neural signals and translate them into control commands. However, non-invasive systems struggle from high levels of noise that make it difficult to extract signals, leading to high error rates and poor performance which can frustrate users.

Given the inherit limitations of non-invasive devices, BRI systems can benefit greatly from shared autonomy architectures. By leveraging the complementary strengths of human cognitive abilities and robotic task automation, BRIs are effective at accomplishing tasks that would be challenging for solely robot or human groups to complete. The level of autonomy has been shown to have significant effects on the comfort and trust users feel toward the system. For systems with little user autonomy, users have reported concerns for safety, with loss of trust once observing failures (Bhattacharjee et al., 2020; Nesset et al., 2023). On the other hand, systems that place too much control in the hands of the user can impose a high cognitive workload, leading to fatigue and reduced long-term usability (Hinss et al., 2022). Despite these known tradeoffs, most PAR papers lack a comparison between levels of autonomy in order to find out what is the most appropriate for users.

Therefore, there is a need for a BRI system that (1) minimizes social isolation by enabling users to interact and collaborate with other humans and robots, (2) evaluates distinct implementations of shared autonomy to understand how specific design choices impact the trade-off between control and error tolerance in ADL tasks, and (3) provides task versatility through a robust framework capable of generalizing across a variety of ADL tasks.

In this work, we extend the M4Bench platform (Yoshida et al., 2025) into a multi-human, multi-robot collaborative system designed to meet these criteria. Our system enables users with mobility impairments to teleoperate a mobile manipulator through a non-invasive BRI, allowing them to cooperatively perform a variety of tasks in a kitchen environment. In the simulated environment, users can move the robot around, pick and place common objects like dishware, glassware, and cookware, but potentially also open and close cabinets and turn appliances on or off.

To understand how the level of autonomy affects usability, we implemented separate control schemes with progressively higher levels of autonomy: Assisted Teleoperation, Shared Autonomy, and Full Automation. We conducted a user study (*n* = 30) and analyzed task performance, workload, ease of use, and sense of agency across the multiple autonomy settings. We found that Full Automation was rated significantly better for workload, ease of use, and speed, whilst Assisted Teleoperation was rated significantly worse for performance, ease of use and sense of agency; in short, users preferred higher levels of automation. Given that our Shared Autonomy mode was as performant as Full Automation and similarly preserved user agency in our design, we believe this indicates that it is a promising paradigm to develop further to allow users to perform tasks of their own volition in more open-ended settings, rather than continuing to rely on more primitive teleoperation methods.

## 2 Related works

### 2.1 PARs

PARs have long been developed to help individuals with disabilities perform ADLs, with the goal of enhancing independence and quality of life. Research has shown that the value of these technologies extends beyond task completion. They can empower users by providing a greater sense of control, reducing feelings of neediness, and enabling more time alone. While specialized devices like the JACO arm have found commercial success for specific tasks such as eating and opening doors (Campeau-Lecours et al., 2016), more general-purpose platforms such as the PR2 (Bohren et al., 2011) and HOBBIT (Fischinger et al., 2016) have been explored for a wider range of household chores. Building on these efforts, NOIR extends such platforms by incorporating EEG, positioning itself as a general-purpose assistive system for ADLs (Zhang et al., 2023).

However, user studies have consistently highlighted significant barriers to adoption, including the robots' slow speed, large size, and a broader perception that the technology is not yet mature enough for reliable home use (Sørensen et al., 2025). Therefore, it is important to develop assistive systems that reduce the physical and cognitive demands on users while maintaining meaningful control for real world adoption.

### 2.2 Shared autonomy

To reduce the physical and cognitive burden on users, shared autonomy has become the dominant control paradigm in assistive robotics. This approach blends control inputs from the human with the robot's autonomous policy, leveraging the human's high-level intent and the robot's precision. However, this introduces a fundamental trade-off. Foundational work by Berberian et al. (2012) systematically demonstrated that increasing levels of automation reduces the sense of agency (SoA), the feeling of being the author of one's own actions. More recently, Collier et al. (2025) extended this finding to assistive robotics, showing an inverse relationship between robotic assistance and SoA. Their results revealed that while greater autonomy reliably improved task success and efficiency, it diminished users' subjective sense of control. These observations highlight SoA as a critical but often overlooked metric in the design and acceptance of assistive systems.

Despite evidence that users value different levels of autonomy in different contexts, most PAR studies only evaluate a single level (Nanavati et al., 2023). In this study, we address this gap by directly investigating the SoA-performance trade-off across distinct autonomy levels in a physically assistive environment.

### 2.3 Noninvasive BRIs

For individuals with severe motor impairments, BRIs offer a powerful alternative for control. These systems create a direct communication pathway from the brain to an external device. Several non-invasive methods are commonly used. Motor imagery (MI) is an endogenous paradigm in which the user imagines a movement, producing detectable changes in sensorimotor rhythms in EEG recordings (Pfurtscheller et al., 2003). While MI is intuitive and closely mirrors natural motor planning, it often requires substantial user training to achieve reliable control (Hayta et al., 2022).

In contrast, exogenous paradigms rely on external stimuli to evoke brain responses. Steady-state visually evoked potentials (SSVEP) exploit the brain's response to flickering lights, allowing rapid selection of a small set of commands (Sutter, 1992). The P300 potential, triggered using an “oddball” paradigm, is well-suited for selecting from a larger set of discrete options (Farwell and Donchin, 1988). Comparative studies have highlighted trade-offs between these approaches in terms of speed, accuracy, and user comfort (Zhao et al., 2015). For example, SSVEP can be physically uncomfortable or visually fatiguing for extended use, while P300 setups can be cumbersome or slow for frequent interactions.

Here, we chose to use MI as the control paradigm because it provides the most generalizable mapping to real-world tasks, despite the additional training required. This choice prioritizes long-term usability, aligning with our goal of designing assistive systems that can be effectively integrated into daily life.

### 2.4 Collaborative assistive systems

While BRIs and PARs have demonstrated the potential to support individual users in performing ADLs, most research has focused on single-human, single-robot interactions (Gordon et al., 2023). In practice, many real-world tasks could benefit from multiple users or multiple robots working together. Multi-human, multi-robot collaboration can reduce the cognitive and physical load on individual users, allow parallel task execution, and enable assistance for more complex ADLs. Existing frameworks for multi-human, multi-robot collaboration have been developed in domains such as rescue (Williams, 2020) and manufacturing (Duan et al., 2024), but their application to assistive technology in home environments remains limited.

**Figure 1:**
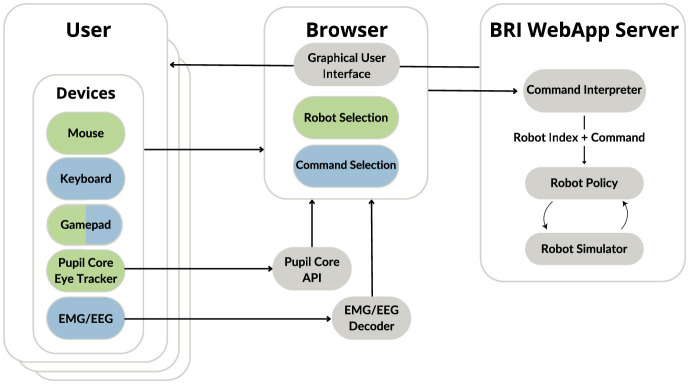
Overview of the M4Bench framework. The platform enables multi-user (signified by the stacked “User” boxes) and multi-robot interactions in a shared environment. Figure reproduced from Yoshida et al. (2025) with permission from the authors.

To address this gap, we previously introduced M4Bench (Figure 1), a testbed for evaluating multi-user, multi-robot assistive interactions (Yoshida et al., 2025). The initial version implemented a simple cube sorting task with fixed-arm manipulators, providing a controlled environment to study shared control strategies with multiple input modalities. This work, however, represents a major overhaul of the software to produce a more generalizable middleware layer for integrating multiple users, multiple robots, and multiple input modalities all in a shared and realistic virtual environment. It now supports more complex multi-task environments, adds mobile manipulators, incorporates machine-learning-based robot control policies, and has an improved user interface. Overall, this framework unifies perception, biosignal-based intent decoding, and shared control in a reusable structure that primarily enables a systematic exploration of autonomy allocation across participants and agents, as well as generalist robot manipulation policies. Moreover, its modular structure and event-based design of the various biosignal-based modalities make this platform easy to extend with new input devices and decoding paradigms, new generalist robotic manipulation policies, and simulated environments that closely mimic real-world scenarios in the context of assistive living.

## 3 Materials and methods

### 3.1 System overview

**Figure 2:**
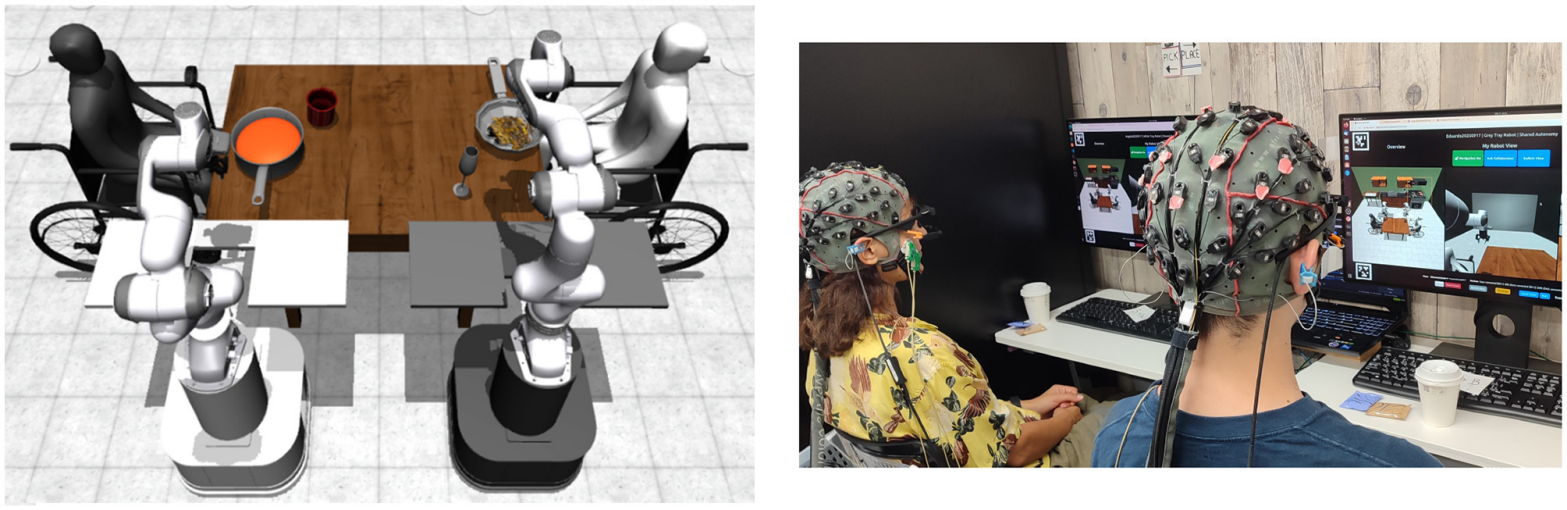
Overview of our system. **(Left)** View of the kitchen environment with mobile manipulators having placed food and drinks in front of the users in simulation. **(Right)** Participants interacting with the shared environment on separate workstations.

Our setup consists of two users and two mobile manipulator robots operating in a shared kitchen environment (Figure 2). Each user controls one robot through a BRI that displays a live video feed from the robot's camera. The goal is to enable collaborative task execution by controlling their respective robot.

The system integrates three complementary input modalities to interact with the UI for flexible robot control: eye tracker, EEG and EMG. The eye tracker monitors the user's gaze and is used to interact with the user interface and to focus on object of interest. EEG signals, decoded from motor imagery tasks, are used to choose among the available actions for the robot. EMG signals serve as a safety mechanism by detecting a jaw-clench pattern that allows the user to cancel a selected action at any time prior to execution.

### 3.2 Levels of autonomy

The system is designed to be flexible and adaptable, supporting configurable levels of shared autonomy. This allows the degree of control between the user and the robot to be adjusted to suit different tasks or user preferences.

**Level 1—Assisted Teleoperation (AT):** Users maintain near-complete control over the task. They provide manual input for navigation using eye-tracking commands, select objects via eye tracking, and issue action-level commands using EEG.^2^ At this level, the robot acts primarily as an executor of detailed instructions.

**Figure 3:**
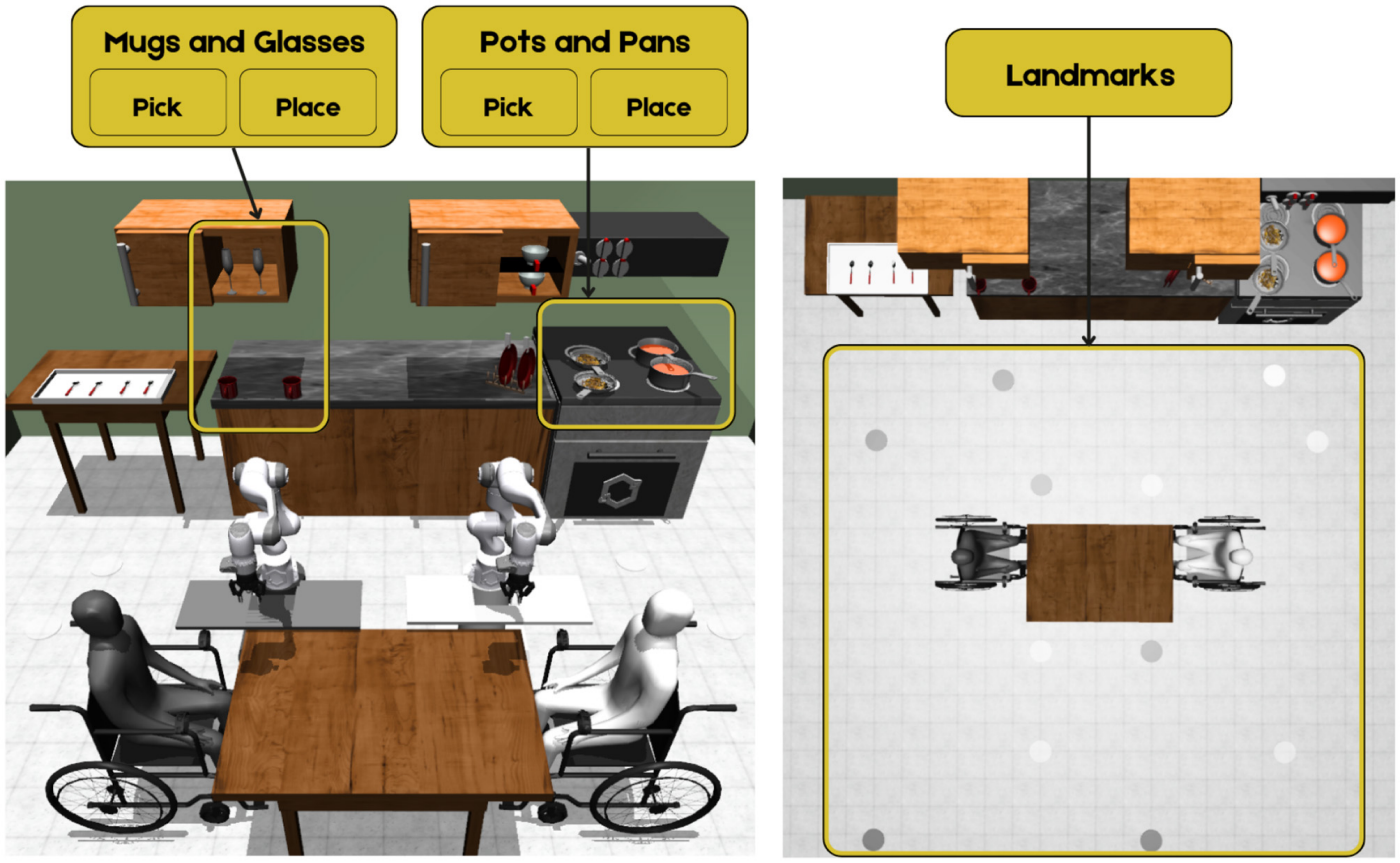
**(Left)** View of the kitchen environment, showing the objects and tasks the robot can carry out in this study. **(Right)** Top view of the environment, highlighting the landmarks each robot can traverse identified by color.

**Level 2—Shared Autonomy (SA):** At this level, the robot takes on part of the workload, reducing the need for step-by-step control. Users still select objects via eye tracking and issue high-level commands with EEG, similar to AT (level 1). The main difference is navigation: users simply select a landmark (Figure 3 Right) with the eye tracker, and the robot moves autonomously, allowing them to focus on higher-level decisions.

**Level 3—Full Automation (FA):** Users communicate only their overall intention, such as selecting a food or drink item using EEG, and the robot autonomously carries out navigation and manipulation. In this condition, user input is minimal, focusing on high-level goal selection rather than stepwise control. Due to the high-level action selection, only one input device is used, which can affect the overall efficacy of the system.

We note that while level 3 might appear convenient, it limits the open-endedness of the system as the meal choices and action sequences need to be pre-defined.

### 3.3 Environment

We built a kitchen environment using the RoboHive framework (Kumar et al., 2023), borrowing assets from the Franka Kitchen environment (Gupta et al., 2019), with MuJoCo (Todorov et al., 2012) as the underlying physics simulator. The environment features two wheelchair users and two mobile manipulators operating within a simulated kitchen setting. The kitchen is equipped with common household items and appliances typically found in real-world homes, including a stove, countertop, cabinets, and a dining table. The assets for these objects were taken from publicly available repos and websites^3^ or datasets, i.e., YCB (Calli et al., 2015) and MetaFood3D (Chen et al., 2024), chosen for being derived from real-world objects.

The variety of objects and tools available in the environment allows users to instruct the robots to perform a wide range of actions and tasks (Figure 3 Left). The environment is designed to simulate a mealtime scenario, which involves multiple activities such as meal preparation, table setting, assisted eating and drinking, and post-meal clean-up.

### 3.4 Assistive robots

Each robot comprises of a 7-DoF Franka Emika Panda arm mounted on a holonomic mobile base and equipped with a Robotiq 2F-85 gripper. The Franka robot is a collaborative robot designed to operate safely in the same workspace as humans, capable of lifting various objects required for household tasks. The mobile base is based on TidyBot2 (Wu et al., 2024), an open-source hardware robot that can be built from commercially available parts. This holonomic base allows the robot to move in any direction and rotate on the spot, simplifying navigation between predefined task station locations and enabling more compact maneuvering in constrained kitchen environments.

The mobile base is equipped with RGBD cameras that provide the robots with both color and depth information about their surroundings. Each arm is equipped with a front-facing camera that provides a real-time egocentric view, which is streamed directly to the user interface.

### 3.5 Robot control

Each manipulator is mounted on a holonomic mobile base. For manipulation tasks, we adopt imitation learning techniques rather than classical trajectory planners: while the latter requires building an accurate model of the environment—infeasible at scale—the former allows the robot to learn directly from human demonstrations with few assumptions about the task or environment. In contrast, the navigation part is not learned but handled by a standard path planner.

We collected 70 demonstrations of each manipulation task using human teleoperation with a gamepad. A deep learning model inspired from RISE (Wang et al., 2024) was trained using supervised learning to mimic these human demonstrations. The model takes as input an RGB point cloud of the scene reconstructed using the robot onboard cameras, and it is trained using a flow matching objective (Lipman et al., 2022) to output the desired end-effector command.

In a kitchen environment, the robot might need to extend to reach high shelves or objects placed at the back of counters. To maintain safety during robot operation, we use the operational space control barrier function (OSCBF) framework (Morton and Pavone, 2025). With negligible computational cost, via OSCBF we constrain the robot's motion to remain well-behaved and within safe bounds, maintaining reliable and predictable behavior even in critical situations. Notably, neither the imitation learning policy nor OSCBF require privileged information. These design choices guarantee that every component used for manipulation can be deployed on a real robot without modifications.

During deployment, the robot was allocated a maximum of 30 seconds (300 environment steps in simulation) to complete the requested manipulation task (e.g., picking up a pot of pasta from the stove and placing it on the tray). If the robot failed to complete the subtask within this window, it was recorded as a failure. The arm was then returned to its home configuration, and the manipulated object was reset to be within the distribution of the training data used to train the manipulation policy. While this introduces some simplification compared to reality, it minimizes the influence of robot failures on the user experience by compensating for cases in which objects were displaced during unsuccessful attempts. In instances where the user issued an infeasible command (e.g., requesting the robot to place the pasta on the table before it had been grasped), the system ignored the command to prevent cascading errors.

#### 3.5.1 Manipulation reset protocol

We implemented different recovery behaviors corresponding to the different autonomy levels when manipulation attempts fail. After each failure, both the object and the robot are reset, and the user–robot interaction proceeds as follows:

At AT (level 1), after each failed attempt the user must manually repeat both the eye tracker selection and the motor imagery command before the robot can try again.

At SA (level 2), the robot automatically retries the manipulation once after the first failure. If the second attempt also fails, the user is required to repeat the eye tracker selection and motor imagery command.

At FA (level 3), the robot continues to retry the manipulation autonomously until the action succeeds or the episode times out, with no additional input from the user.

### 3.6 User interface

**Figure 4:**
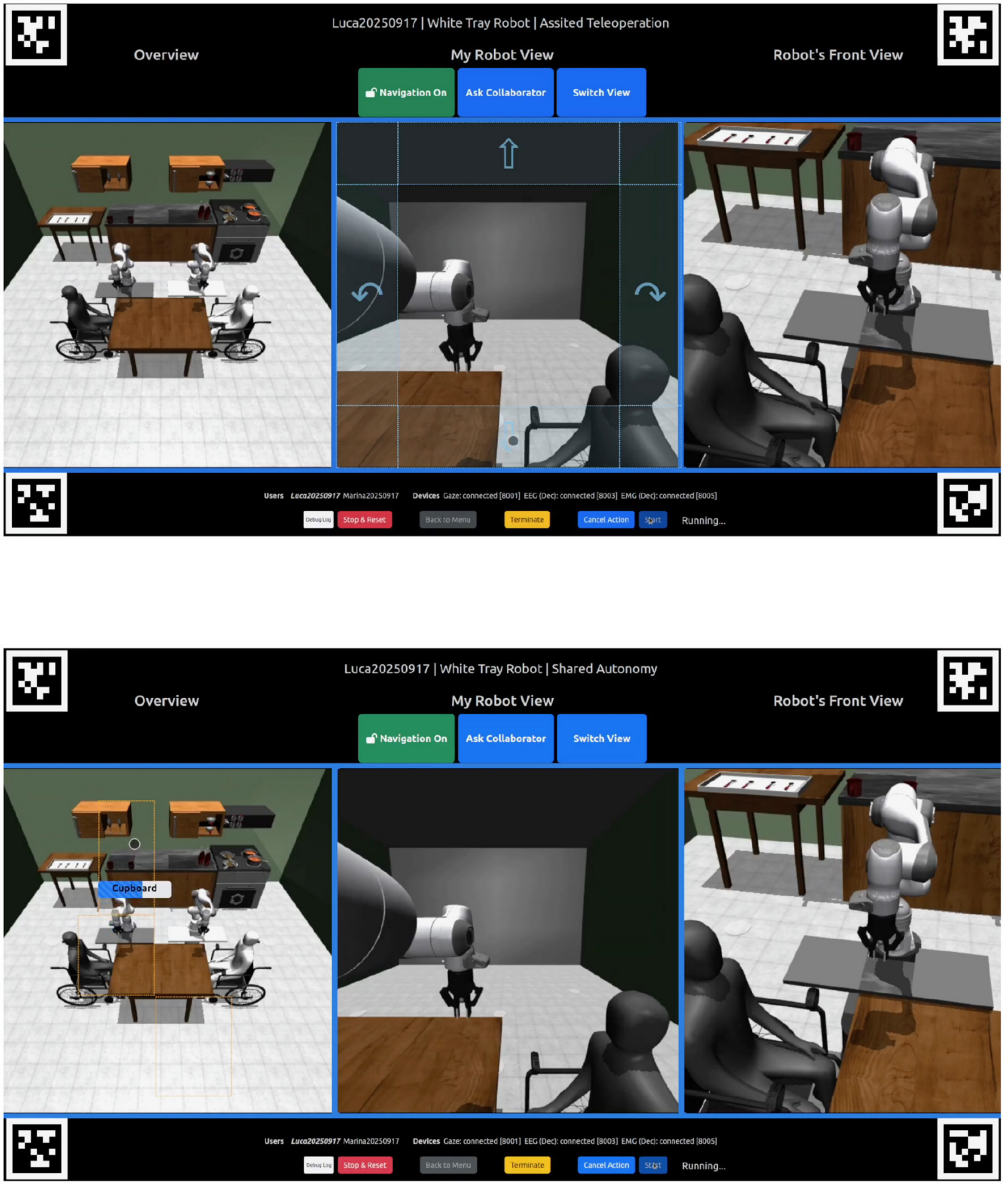
User Interface at different autonomy settings with their different navigation cues. **(Top)** User Interface at the Assisted Teleoperation level. In the central view the blue shaded areas represent the directional commands the user can use to move the robot within the environment by focusing on them. **(Bottom)** User Interface at the Shared Autonomy level. In the leftmost view, dotted orange boxes indicate the predefined landmarks to which the user can send the robot by focusing on them. In this example, the user is focusing on the Cupboard landmark.

The user interface consists of three primary views: *Overview, My Robot View*, and *Robot's Front View* (Figure 4). The *Overview* includes all entities in the environment, including the 2 users, 2 robots, table and kitchen area. This view serves as a global reference map, so the user has a constant understanding of where each robot is relative to its surrounding environment. *My Robot View* shows an egocentric perspective of the robot assigned to the user, enabling more precise interactions with the environment. *Robot's Front View* provides a front-facing perspective of the robot, giving users an additional external view to monitor robot movements and spatial relationships.

In addition to the three main views, the interface included three toggle buttons that supported navigation control and collaboration (Figure 4). The *Navigation On-Off* toggle allowed users to temporarily disable navigation commands, preventing unintended robot movements when focusing on object selection in *My Robot View* or *Overview* in levels 1 and 2. This was particularly useful when navigation controls were positioned close to objects, reducing the risk of accidental robot movement during selection.

**Figure 5:**
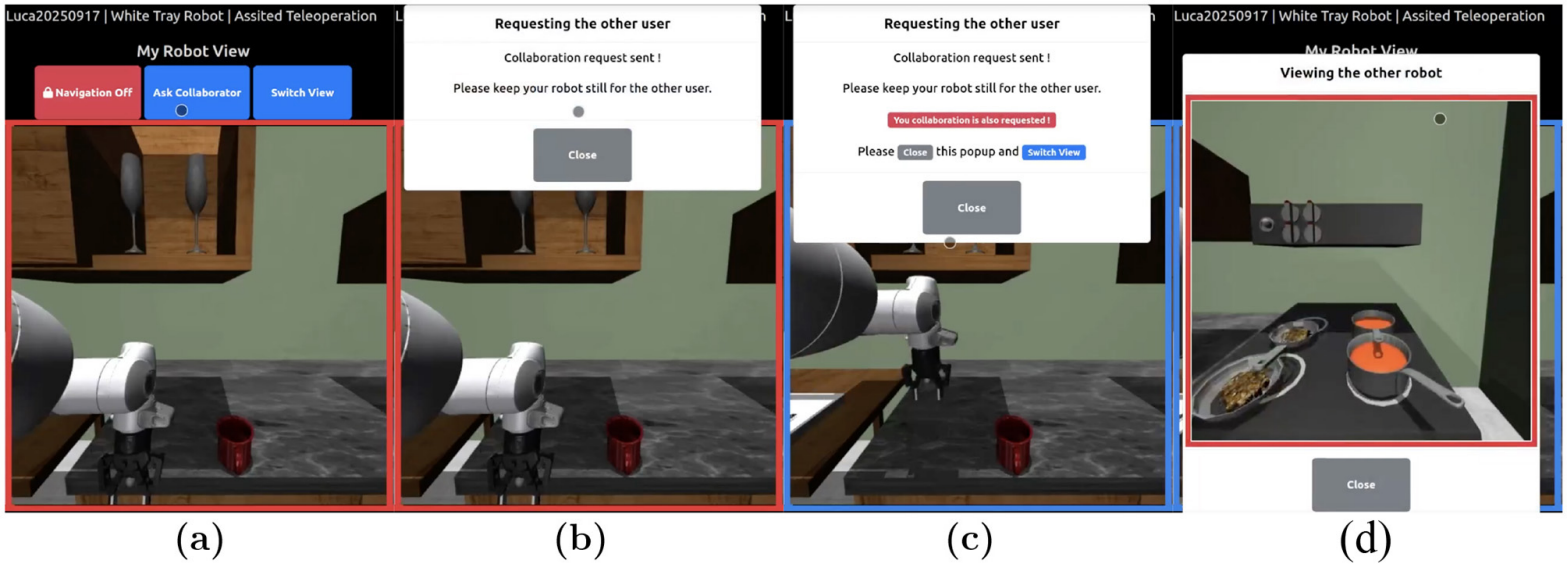
Collaboration request work flow: **(a)** User 1 sends a request to control User 2's robot. **(b)** User 1 waits for User 2 to accept the collaboration request. **(c)** User 1 is notified that the collaboration has been approved. **(d)** User 1 controls User 2's robot to complete the action.

The *Ask Collaborator* toggle enabled users to actively request assistance from their partner during task execution (Figure 5). When a participant selected an item for their assigned robot, pressing this button sent a request to the other user, signaling that intervention was needed. The partner is then required to accept the request for the collaboration to take place. The recipient could then temporarily take control of the first user's robot via the *Switch View* toggle to complete the required action. Once the task was finished, control automatically returned to the original user. This feature facilitated efficient collaboration, ensuring that both participants could coordinate their actions and complete shared tasks effectively, particularly in AT (level 1) and SA (level 2) where user input is essential for robot operation.

#### 3.6.1 User interaction for robot control

The interface integrated three input modalities—eye tracking, EEG, and EMG—each mapped to a distinct function. Eye tracking was used for robot navigation and for selecting objects within the environment. EEG served as the mechanism for action selection once an object had been chosen, while EMG provided a cancellation function to override erroneous EEG selections.

Navigation was implemented across two levels. With AT (level 1), directional commands were presented in *My Robot View*, allowing users to move the robot forward, backward, or rotate in place by fixating on the corresponding zones (Figure 4 top). When the robot approached a landmark, it automatically docked to the location. At SA (level 2), navigation was performed in *Overview*, where users could move the robot to predefined landmarks such as the table sides, the food zone, or the drink zone (Figure 4 bottom). In this mode, low-level navigation was automated, and the robot followed pre-defined paths connecting intermediate waypoints to reach the selected landmark.

**Figure 6:**
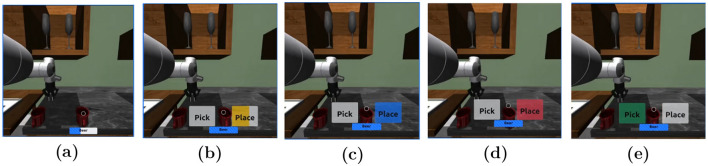
Examples of the user interface during manual task execution. **(a)** The progress bar fills as the user fixates on an item using the eye-tracker. **(b)** Two options are displayed, and the user's MI gradually fills the intended choice in yellow. **(c)** Once the option is selected, it turns blue, indicating the confirmation phase and allowing the user to apply an EMG-based correction if needed. **(d)** If the user performs a jaw clench, the selected option turns red to indicate cancellation. **(e)** The alternative option is then automatically executed and turns green to confirm the final selection. In case the user's intent was properly decoded via EEG, the correct option turns green before being executed.

During manual task execution (levels 1, 2), object selection was achieved via eye tracking. We created a list of objects that the users can interact with and used the open vocabulary OWLv2 object detector (Minderer et al., 2024) to dynamically find and construct bounding boxes for the listed objects from the robot camera view. When a user's gaze hovers on an object, an empty progress bar with the object type in the center is displayed. The progress bar then progressively fills as the user maintains focus on said object for up to 3 seconds (Figure 6a). Once the progress bar is filled, the object is considered as selected, and two additional progress bars are shown on the left and right side of the gaze cursor, each side labeled with the corresponding action (Figure 6b). To prevent bounding boxes remaining on screen we implemented a timeout of 10s.

EEG was used to select between two possible actions. When users performed MI corresponding to their intended choice, the associated option first turned blue, and then turned green to indicate confirmation. If users wanted to correct the selection, they could activate the EMG by clenching their jaw while the option was still blue. In that case, the selected option turned red, and the robot automatically executed the alternative action, which turned green to confirm the command. If no EMG activity was detected, the initially chosen option simply turned green as confirmation (Figure 6).

**Figure 7:**
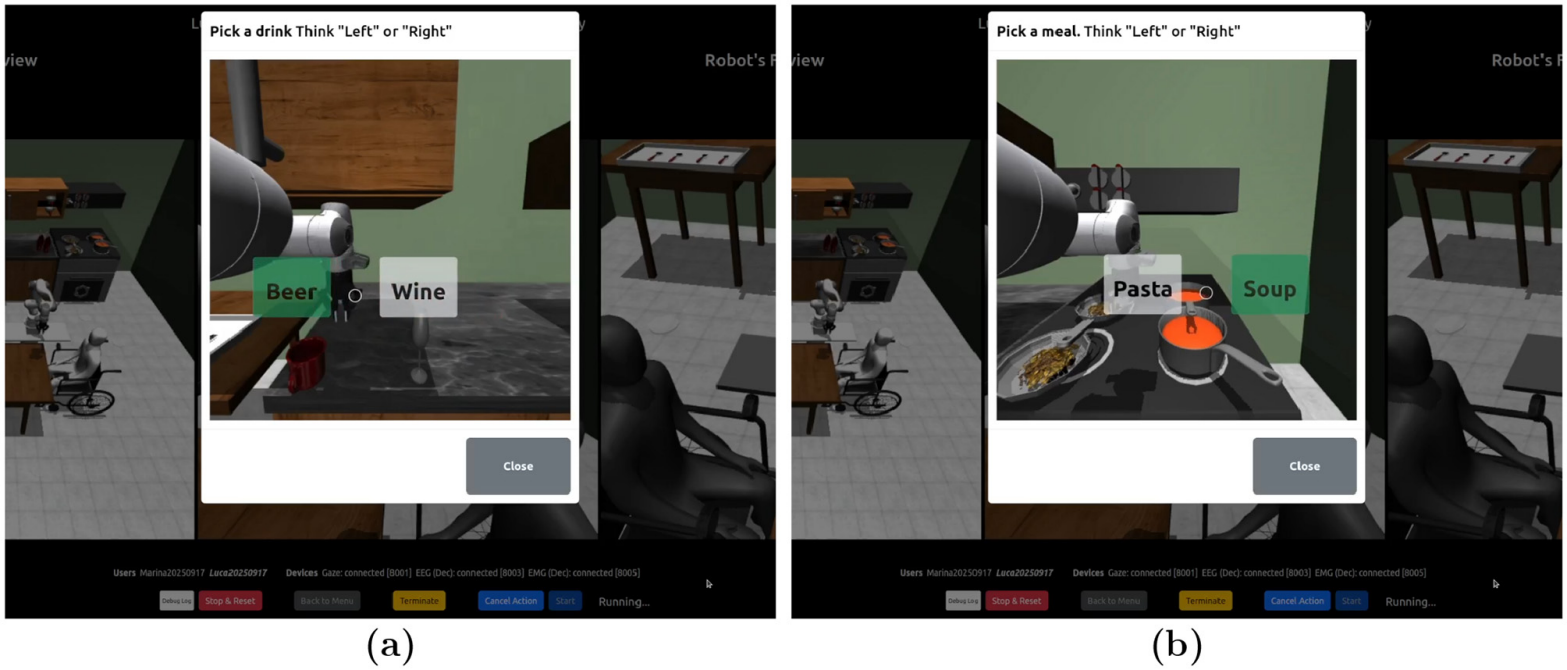
User interface for FA (level 3). Each user chooses their drink **(a)** and meal **(b)** using MI.

With FA (level 3), users receive a sequence of camera screenshots of interactive areas, and must choose between the objects (Figure 7). The possible objects are predefined and not detected by the object detector and eye tracker as in levels 1 and 2. Implicitly this also dictates the action sequences (e.g., pick and then place). Whilst this is possible under relatively controlled settings, this mode does restrict the open-endedness of the system, as the sequence of actions must be set in advance. In addition, this also removes explicit collaboration from the task.

### 3.7 User input devices

#### 3.7.1 Eye tracker

Eye gaze data was collected using the Pupil Labs Pupil Core eye tracker.^4^ The device continuously recorded the user's gaze position on the display, providing x–y screen coordinates in real time. It is possible to wear the eye tracker on top of glasses, enabling visually-impaired users to also use this system.

#### 3.7.2 EEG

EEG data was recorded using a 64-channel g.tec g.GAMMAcap2 system with active g.SCARABEO electrodes^5^ arranged according to the extended International 10–20 system. For motor imagery decoding, we focused on 22 electrodes positioned over sensorimotor regions (FZ, FC3, FC1, FCZ, FC2, FC4, C5, C3, C1, CZ, C2, C4, C6, CP3, CP1, CPZ, CP2, CP4, P1, PZ, P2, and POZ). A1 served as the reference electrode, and AFZ was used as the ground. Signals were sampled at 256 Hz, and electrode impedances were maintained below 5 kΩ throughout data collection to ensure high signal quality.

Given that EEG data was acquired simultaneously from two participants (hyperscanning) in the same room, we adopted precautions to address the risk of technical artifacts and spurious signal correlations as reported by Burgess (2013). Such artifacts can emerge from shared environmental noise, electrical coupling, or cable cross-talk (Zamm et al., 2024). To mitigate these potential confounds and ensure data integrity, we implemented several hardware and environmental controls, following established hyperscanning hardware practices (Barraza et al., 2019) and best-practice recommendations (Zamm et al., 2024). All recording equipment and power outlets were properly grounded, and conductive grounding mats were placed under each participant and near main cable junctions to reduce electromagnetic interference. EEG cables were carefully routed to maintain physical separation and avoid parallel runs, thereby minimizing the risk of capacitive or inductive coupling. Each EEG cap was operated independently, with its own dedicated reference (A1) and ground (AFz) electrodes, preventing artifacts arising from shared reference paths. Finally, participants were positioned to minimize shared sensory cues (e.g., incidental visual stimuli), further reducing potential sources of non-neural signal correlation.

#### 3.7.3 EMG

EMG recordings were collected using two passive g.tec EMG electrodes (plus one ground electrode) placed on the left masseter muscle with a reference electrode placed on clavicular head of the sternocleidomastoid muscle. The signal consists of one channel, formed by subtracting the anode potential from the cathode potential.

### 3.8 Biosignal decoders

#### 3.8.1 Eye tracker

The Pupil Eye Tracker software was used to record and decode real-time x-y gaze coordinates on the screen, enabling user interface interactions. To improve stability and reduce rapid fluctuations in gaze signal, the eye tracking data was smoothed by calculating a moving average over the latest 35 API samples before use in experimental tasks.

#### 3.8.2 EEG

A calibration session was conducted prior to online decoding, during which each participant completed 10 min of MI tasks. The calibration consisted of 96 trials, each including a visual cue (500 ms), an MI period (4 s), and a rest period (500 ms). During the visual cue, a left or right arrow was displayed to indicate the upcoming motor imagery period and then disappeared. The rest period was indicated by a gray screen.

As initial preprocessing, the raw EEG data had a notch filter set at 48–52 Hz to suppress line noise from mains electricity. Additional EEG preprocessing was performed using the MOABB library's (Jayaram and Barachant, 2018) default MI pipeline, including bandpass filtering (8–32 Hz), epoching, and signal standardization.

Feature extraction was performed using the filter bank common spatial pattern algorithm (FBCSP) (Koles et al., 1990; Ang et al., 2008). The EEG signals were decomposed using a bank of 6 bandpass filters spanning the frequency range of 8–32 Hz, which encompasses both alpha (8–13 Hz) and beta (13–30 Hz) rhythms that are particularly relevant for MI. From each frequency band, 4 CSP components were extracted, resulting in a total of 24 features per trial. Classification was performed using a support vector machine (SVM) with a radial basis function kernel and a regularization parameter of *C* = 0.5. The classifier was configured to output probability estimates for the two classes (left vs. right).

To compare offline decoder performance *post hoc*, we implemented two additional MI EEG pipelines: CSP + linear discriminant analysis (CSP + LDA) (Wu et al., 2013) and augmented tangent space + SVM (Aug + Tang + SVM) (Carrara and Papadopoulo, 2024). CSP + LDA was adopted due to its simplicity and validation in previous works (Dillen et al., 2024), while Aug + Tang + SVM was selected to leverage its leading classification accuracy on large-scale datasets (Chevallier et al., 2024). Both pipelines used the same initial preprocessing steps as previously described.

For the CSP + LDA pipeline, 8 CSP components were extracted, which were then classified using LDA. For the Aug + Tang + SVM pipeline, the EEG data was first augmented using the MOABB AugmentedDataset method. The EEG covariance matrices were then computed for each trial. Features were derived from the covariance matrices by projecting them from the Riemannian manifold onto the tangent space, using the Riemannian mean of the data as the reference point, and then vectorizing the matrices. Finally, the features were classified by an SVM with a radial basis function kernel.

For all pipelines, model evaluation used a stratified 5-fold cross-validation strategy implemented with MOABB's WithinSessionEvaluation class. The model achieving the highest mean cross-validation accuracy was selected for online decoding.

During the online phase, EEG data was streamed in real time via the Lab Streaming Layer protocol. Continuous EEG data was segmented into epochs, which were then processed using the same preprocessing and feature-extraction pipeline as in the offline analysis. A sliding-window approach was applied, with a classification triggered only if the same class was decoded three times consecutively. This voting mechanism reduced spurious outputs and increased the stability of online decisions.

#### 3.8.3 EMG

For EMG recordings, we used a single pair of passive g.tec EMG electrodes (plus one ground electrode). The signal consists of one channel, formed by subtracting the anode potential from the cathode potential. We apply a notch filter at 50 Hz to reduce line noise, and a bandpass filter from 2–60 Hz.

To train our EMG decoder, we collect blocks of “rest” and “clench” data of 3s each. The EMG decoding pipeline consists of taking the maximum amplitude of the signal, and then applying a SVM classifier with a polynomial kernel. We use a test set comprising of 20% of the collected EMG data.

## 4 Shared autonomy study

The purpose of this study was to systematically evaluate our multi-modal BRI across different levels of shared autonomy. Shared autonomy has been proposed as a promising paradigm for BRI systems, allowing users to retain meaningful control while offloading lower-level or repetitive tasks to the robot. However, little is known about how varying degrees of autonomy affect user performance, workload, usability, and sense of agency in multi-user BRI contexts.

**Figure 8:**
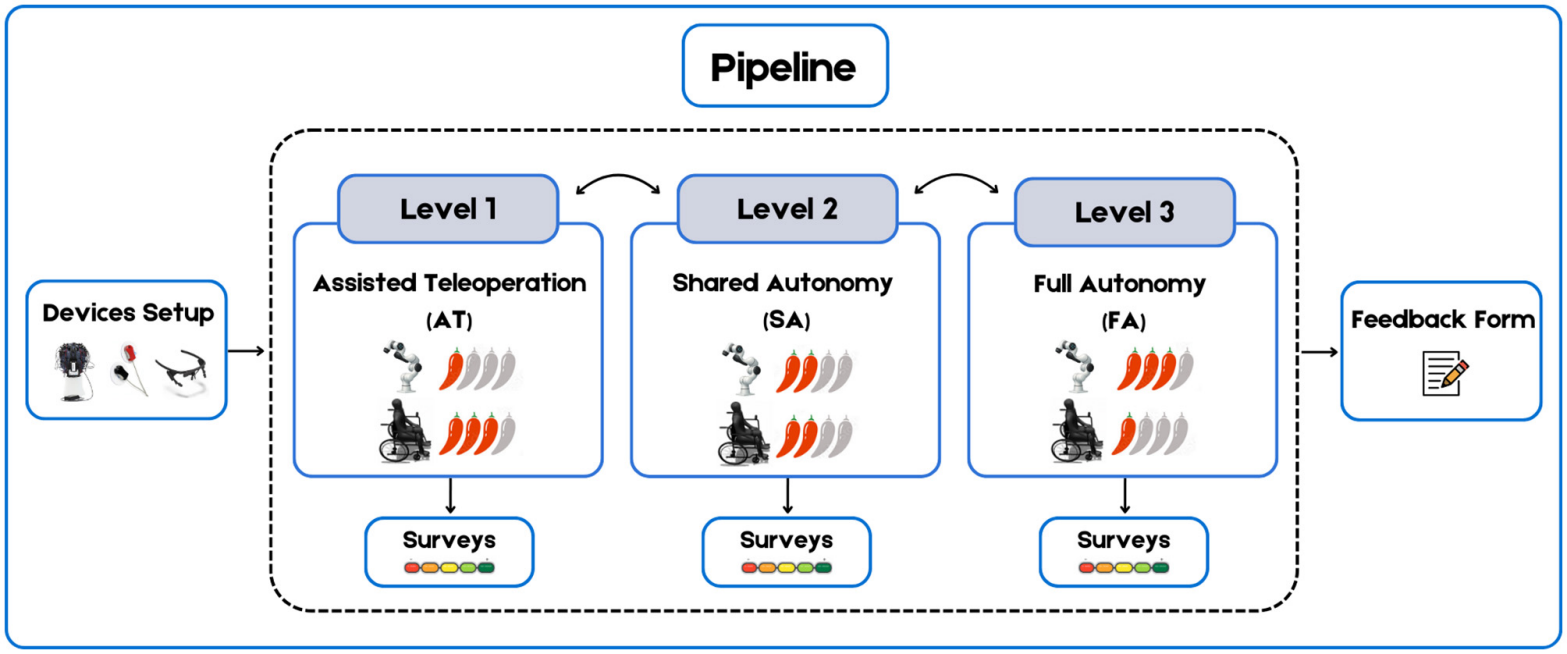
Pipeline of the shared autonomy study. Following device setup, participants received instructions for the specific autonomy level, completed a practice trial, and then performed the collaborative task under the three randomized autonomy levels (Assisted Teleoperation, Shared Autonomy, and Full Automation). After each level, participants completed the three surveys (SUS, NASA-TLX, and SoARS), and a final feedback form was collected at the end of all sessions.

To address this gap, we conducted a controlled study with 30 healthy participants (15 pairs; 9 females; age 30.9 ± 6.7 years), who jointly operated two mobile manipulator robots in a simulated kitchen environment. Across repeated sessions, participants completed the same collaborative task under three different levels of robot autonomy: Assisted Teleoperation, Shared Autonomy, and Full Automation (Figure 8). The order of autonomy levels was randomized to control for sequence effects.

Participants were first briefed on the purpose of the study and provided instructions on the task and the input devices. Written consent was obtained prior to the experiment, after which the setup was completed and trials commenced. Our study was approved by the Shiba Palace Clinic Ethics Review Committee.

We focused on four primary research questions:

1. **Performance:** How does the level of robot autonomy affect participants' ability to successfully complete tasks?
2. **Workload:** In what ways does shared autonomy influence the physical and mental workload reported by participants?
3. **Ease of use:** How intuitive do participants find the system at different autonomy levels?
4. **Agency:** To what extent do users feel a sense of control and authorship over the robot's actions under varying autonomy conditions?

We evaluated human, robot, and system metrics (Steinfeld et al., 2006) to obtain a comprehensive assessment of performance and user experience.

### 4.1 Task

The task took place in a simulated kitchen environment containing two users, two mobile manipulator robots, and four interactive items (two food options and two drink options). In the simulation, each user was seated on either side of the table and controlled one robot assigned to them. For each session, both users were assigned a food and drink to serve themselves—for example, one user could be assigned to serve themselves pasta and wine. The task was considered complete when both users had the correct items placed on the table in front of them. A task was considered failed if an incorrect item was delivered or if all required items were not placed within the 10 min time limit.

During each trial, participants instructed their assigned robot to retrieve the designated items and place them on the table. In order to accomplish this, the user-robot pair must navigate to a predefined landmark (stove, cabinet, or tableside) and perform a manipulation task (e.g. pick up pasta). Manipulation actions were only permitted once the robot had reached one of these landmarks. Because each robot could only retrieve either drinks or food, collaboration between users was required. When a participant is assigned the food robot, they would first pick their own item (e.g., pasta). They would then use the Ask Collaborator feature to request assistance from the other user, who could temporarily control their robot to select the drink item (e.g., wine) for themselves. Participants then regained control of their robot to deliver both items to the correct locations on the table.

Across sessions, the task itself remained constant, but the amount of control the user had over the robot differed, reflecting the three levels of autonomy. Differences in how the task was performed at each autonomy level are summarized in Table 1. Failed manipulation attempts were handled according to level-specific recovery behaviors (Section 3.5.1). This design allowed us to evaluate how varying autonomy levels influenced task performance and user experience.

**Table 1:** Summary of task workflow across the three levels of autonomy.

**Task stage**	**Level 1—AT**	**Level 2—SA**	**Level 3—FA**
Input modality	Eye tracker, EEG, EMG	Eye tracker, EEG, EMG	EEG, EMG
Object selection	Eye tracker	Eye tracker	EEG
Action selection	EEG	EEG	
Cancellation	EMG	EMG	EMG
Navigation	Manual commands via eye tracker	Landmark selection via eye tracker	-
Collaboration	Control by second user	Control by second user	-

### 4.2 Metrics

To evaluate both the performance and usability of our system, we implemented a set of widely used human-robot interaction (HRI) metrics, categorized into human, robot, and interaction/system metrics (Steinfeld et al., 2006). Additionally, we asked participants two open-ended questions to gather detailed feedback on their experience.

#### 4.2.1 Human metrics

- NASA task load index (NASA-TLX): Subjective workload assessment across six dimensions (mental demand, physical demand, temporal demand, effort, performance, and frustration). An unweighted score is calculated by simply averaging the six dimension ratings. Higher scores indicate greater perceived workload (Hart and Staveland, 1988).
- System usability scale (SUS): A 10-item questionnaire with ratings on a Likert scale from 1–5. To generate the score, a formula is applied by summing the scores for odd-numbered questions, subtracting 5, summing the scores for even-numbered questions, subtracting 25, then summing these results and multiplying by 2.5. The alternating positive and negative wording of the statements helps to prevent response bias. Higher scores indicate better perceived usability (Brooke et al., 1996).
- Sense of agency rating scale (SoARS): Developed to assess individuals' subjective sense of control over actions and their outcomes. It provides a multidimensional measure of agency experience, with ratings on Likert-type scales. Participants' ratings on all items are summed after reverse-scoring negatively worded items so that higher scores reflect a stronger sense of agency. Higher total scores indicate greater perceived control over actions and outcomes. It is also possible to divide SoARS into two weighted factors—sense of positive agency (SoPA) and sense of negative agency (SoNA)—which capture whether users feel in control and if they feel helpless, respectively (Tapal et al., 2017).

#### 4.2.2 Robot metrics

- Robot performance: Policy success measuring the reliability in completing its intended action during a trial. A policy is considered successful if the robot executes the commanded behavior correctly without errors or interruptions.

#### 4.2.3 Interaction/system metrics

- Task success: Whether the participants successfully completed the assigned task using the system.
- Decoder success: Whether the participant's MI was successfully decoded by the EEG decoder.
- Timeout: Whether the task exceeded the 10-min time limit. This accounts for failures in any submodules (eye tracking, EMG, EEG, or robot policies) or user strategies that result in infeasible task configurations.
- Completion time: The duration required to successfully complete the assigned task.
- Interaction time: The time taken by the user to successfully provide a command to the robot, measured from when the robot is idle and ready to receive input.

#### 4.2.4 Open-ended questions

1. Among the three levels of robot autonomy you experienced, which one did you prefer and why? If you had to use such a system at home, which level would you choose?
2. In your opinion, would a person with motor disabilities prefer the same level of autonomy and control over the robot's actions that you chose, or a different one? Do you think they would value being more directly in charge of the actions to feel a stronger sense of independence, or would they prefer the robot to take over more tasks for them? Please explain your reasoning.

## 5 Results

In this section, we present the outcomes of our study, organized according to commonly-used HRI metrics categories.

### 5.1 Human metrics

**Figure 9:**
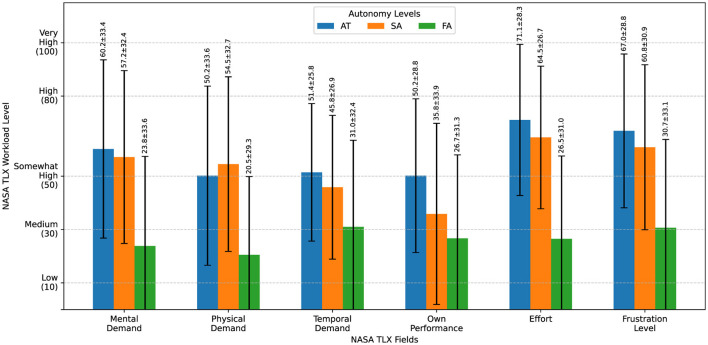
NASA TLX workload evaluation. NASA-TLX workload scores across autonomy levels. Participants reported substantially lower workload in FA (level 3). There was no significant difference in workload between AT (level 1) and SA (level 2).

With NASA-TLX ratings (Figure 9), participants reported higher workload in the manual and shared conditions compared to full automation. After confirming that our data was normally distributed, we ran a repeated measures ANOVA that confirmed a significant effect of autonomy level on NASA-TLX scores [F_(2, 54)_ = 31.85, *p* < 0.001]. *post-hoc* comparisons with Bonferroni correction showed that FA (level 3) resulted in significantly lower workload than AT (level 1) [t_(27)_ = 6.39, *p* < 0.001] and SA (level 2) [t_(27)_ = 6.19, *p* < 0.001]. The difference in perceived workload between AT and SA was not statistically significant [t_(27)_ = 1.65, *p* = 0.33].

**Figure 10:**
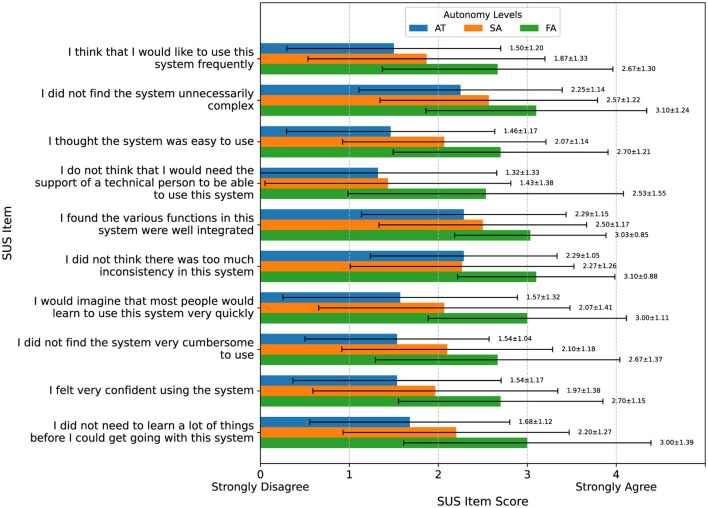
SUS evaluation. Usability improved with increasing automation, from low scores in AT (level 1) to higher scores in SA (level 2) and the highest scores in FA (level 3).

System usability, as measured by SUS (Figure 10, Table A1), improved with as the level of automation increased. After confirming our data was normally distributed, we ran a repeated measures ANOVA that revealed a significant effect of autonomy level on SUS scores [F_(2, 54)_ = 21.10, *p* < 0.001]. *post-hoc* comparisons with Bonferroni correction indicated that FA was rated significantly higher than AT [t_(27)_ = –5.15, *p* < 0.001] and SA [t_(27)_ = –4.60, *p* < 0.001]. Additionally, SA was rated significantly higher than AT [t_(27)_ = –2.64, *p* = 0.04].

**Figure 11:**
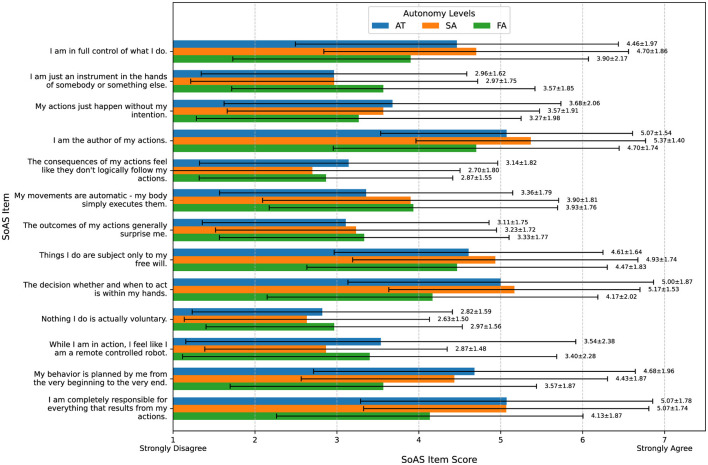
SoARS evaluation. Prospective agency (SoPA) was higher in AT (level 1) and SA (level 2), while FA (level 3) showed increased sense of ownership of navigation (SoNA) and lower SoPA.

Patterns in SoARS scores (Figure 11, Table A2) revealed a significant effect of autonomy on SoPA. After confirming that our data was normally distributed, we ran a repeated measures ANOVA that confirmed a significant effect of autonomy on SoPA [F_(2, 54)_ = 3.70, *p* = 0.046]. However, *post-hoc* comparisons with Bonferroni correction did not yield statistically significant pairwise differences, with the strongest trend observing higher SoPA in SA (level 2) compared to FA (level 3) [t_(27)_ = 2.43, *p* = 0.065]. Differences between AT and SA [t_(27)_ = -1.05, *p* = 0.92] and AT and FA [t_(27)_ = 1.63, *p* = 0.34] were not significant. Autonomy level did not significantly affect SoNA [F_(2, 54)_ = 0.52, *p* = 0.60].

### 5.2 Robot metrics

Policy training success varied across the three autonomy levels tested (Table 2). Success rates under AT (level 1) were generally the lowest, whereas FA (level 3) achieved the highest performance. The lower performance at the AT level can be attributed to users occasionally and unintentionally moving the robot base, which affected task outcomes since imitation learning policies are highly sensitive to initial conditions. In contrast, at the SA and FA levels, users could only command the robot to dock at predefined landmarks, resulting in more consistent initial states that closely matched those seen during data collection for the manipulation tasks. Given the shared docking logic, we anticipate that the performance gap between SA and FA would narrow with increased experimental trials.

**Table 2:** Success rates per policy at each autonomy level, computed over 45 trials and 387 total policy rollouts.

**Policy**	**Assisted Teleoperation**	**Shared autonomy**	**Full automation**	**Overall**
Pick mug	94.7% (18/19)	100.0% (16/16)	68.8% (11/16)	88.2% (45/51)
Pick wine glass	100.0% (12/12)	100.0% (14/14)	94.1% (16/17)	97.7% (42/43)
Pick pasta	58.8% (10/17)	62.5% (15/24)	100.0% (16/16)	71.9% (41/57)
Pick pot	68.0% (17/25)	61.9% (13/21)	87.5% (14/16)	71.0% (44/62)
Place mug	62.5% (10/16)	92.9% (13/14)	73.3% (11/15)	75.6% (34/45)
Place wine glass	80.0% (4/5)	66.7% (10/15)	93.8% (15/16)	80.6% (29/36)
Place pasta	83.3% (5/6)	63.2% (12/19)	72.7% (16/22)	70.2% (33/47)
Place pot	46.7% (7/15)	75.0% (12/16)	86.7% (13/15)	69.6% (32/46)
Overall	72.2% (83/115)	75.5% (105/139)	84.2% (112/133)	77.5% (300/387)

Individual policies also showed variability: for example, the Pick Wine Glass policy reached near-perfect execution (97.7%), while the Pick Pasta policy performed more moderately (71.9%). This difference is mainly due to the different object shapes and weight distributions, requiring different levels of end-effector placement accuracy to achieve a stable grasp.

### 5.3 Interaction/system metrics

Task success varied substantially across the three autonomy levels (Table 3). AT (level 1) yielded the lowest success rate (13.3%, 2/15), SA (level 2) achieved the highest success rate (80.0%, 12/15), and FA (level 3) had a moderate success rate (66.7%, 10/15). A repeated measures ANOVA confirmed a significant main effect of autonomy on task success [F_(2, 28)_ = 14.52, *p* < 0.001]. *post-hoc* comparisons with Bonferroni correction revealed that success rates in SA were significantly higher than in AT [t_(14)_ = 5.29, *p* < 0.001], and success rates in FA were significantly higher than in AT [t_(14)_ = 4.00, *p* = 0.004]. The difference between SA and FA was not statistically significant [t_(14)_ = 1.00, *p* = 1.00].

**Table 3:** Aggregated metrics at task-time, computed over 45 trials (3 trials per user pair with 15 pairs of users).

**Autonomy level**	**Success rate**	**Timeout rate**	**Avg. completion time (s)**	**Avg. interaction time (s)**
Assisted Teleoperation	13.3% (2/15)	66.7% (10/15)	482.5 ± 50.5	249.5 ± 84.9
Shared Autonomy	80.0% (12/15)	6.7% (1/15)	424.8 ± 104.8	142.9 ± 53.3
Full Automation	66.7% (10/15)	0.0% (0/15)	151.1 ± 14.6	17.1 ± 18.5
Overall	53.3% (24/45)	24.4% (11/45)	315.6 ± 159.2	136.5 ± 159.2

Timeouts were most frequent in AT (level 1, 66.7%, 10/15), fewer in SA (level 2, 6.7%, 1/15), and absent in FA (level 3), reflecting the benefit of partial and full automation in preventing task stalls.

Average task completion time decreased with increasing autonomy. AT (level 1) required the longest time to complete tasks (482.5 ± 50.5 s), SA (level 2) produced moderate completion times (424.8 ± 104.8 s), and FA (level 3) achieved the fastest completion (151.1 ± 14.6 s). After confirming that our data was normally distributed, we ran a repeated measures ANOVA that confirmed a significant effect of autonomy level on completion time [F_(2, 28)_ = 93.76, *p* < 0.001]. *post-hoc* pairwise comparisons with Bonferroni correction showed that SA was significantly faster than AT [t_(14)_ = 3.14, *p* = 0.022], FA was significantly faster than AT [t(14)=16.65, *p* < 0.001], and FA was also significantly faster than SA [t_(14)_ = 9.73, *p* < 0.001].

Interaction time showed a marked reduction with higher robot autonomy levels, reflecting the decreased physical and cognitive input required from the user. Participants in AT (level 1) required the longest interaction time (249.5 ± 84.9 s), SA (level 2) substantially reduced user interaction (142.9 ± 53.3 s), and FA (level 3) minimized interaction time further (17.1 ± 18.5 s). After confirming the data was normally distributed, a repeated measures ANOVA revealed a significant effect of autonomy level on interaction time [F_(2, 28)_ = 166.94, *p* < 0.001]. *post-hoc* comparisons with Bonferroni correction indicated that SA had significantly lower interaction time than AT [t_(14)_ = 7.51, *p* < 0.001], FA had significantly lower interaction time than AT [t_(14)_ = 16.41, *p* < 0.001], and FA had significantly lower interaction time than SA [t_(14)_ = 13.72, *p* < 0.001].

Offline decoding of EEG motor imagery achieved moderate classification performance (mean training/validation accuracy = 0.61 ± 0.12; test accuracy = 0.59 ± 0.11). EMG signals were decoded with much higher reliability (train/validation accuracy = 0.96 ± 0.08; test accuracy = 0.96 ± 0.09).

### 5.4 Open-ended questions

Qualitative feedback revealed clear distinctions in user experience across the three autonomy levels. AT (level 1), with high user control, was valued for the sense of agency it afforded, but participants reported high cognitive effort, particularly when executing EEG-based commands. SA (level 2) offered a balance between control and assistance: participants appreciated the robot's autonomous movement, which reduced strain, though some found the system less engaging due to its automated portions. FA (level 3) was generally preferred for routine tasks, as it minimized cognitive load and effort, though users expressed a desire for additional feedback and the ability to intervene when needed. Across levels, participants emphasized that preferences are task and context dependent, and suggested that hybrid systems enabling gradual adaptation between autonomy and control may best accommodate those with motor disabilities.

### 5.5 Decoder *post hoc* analysis

**Figure 12:**
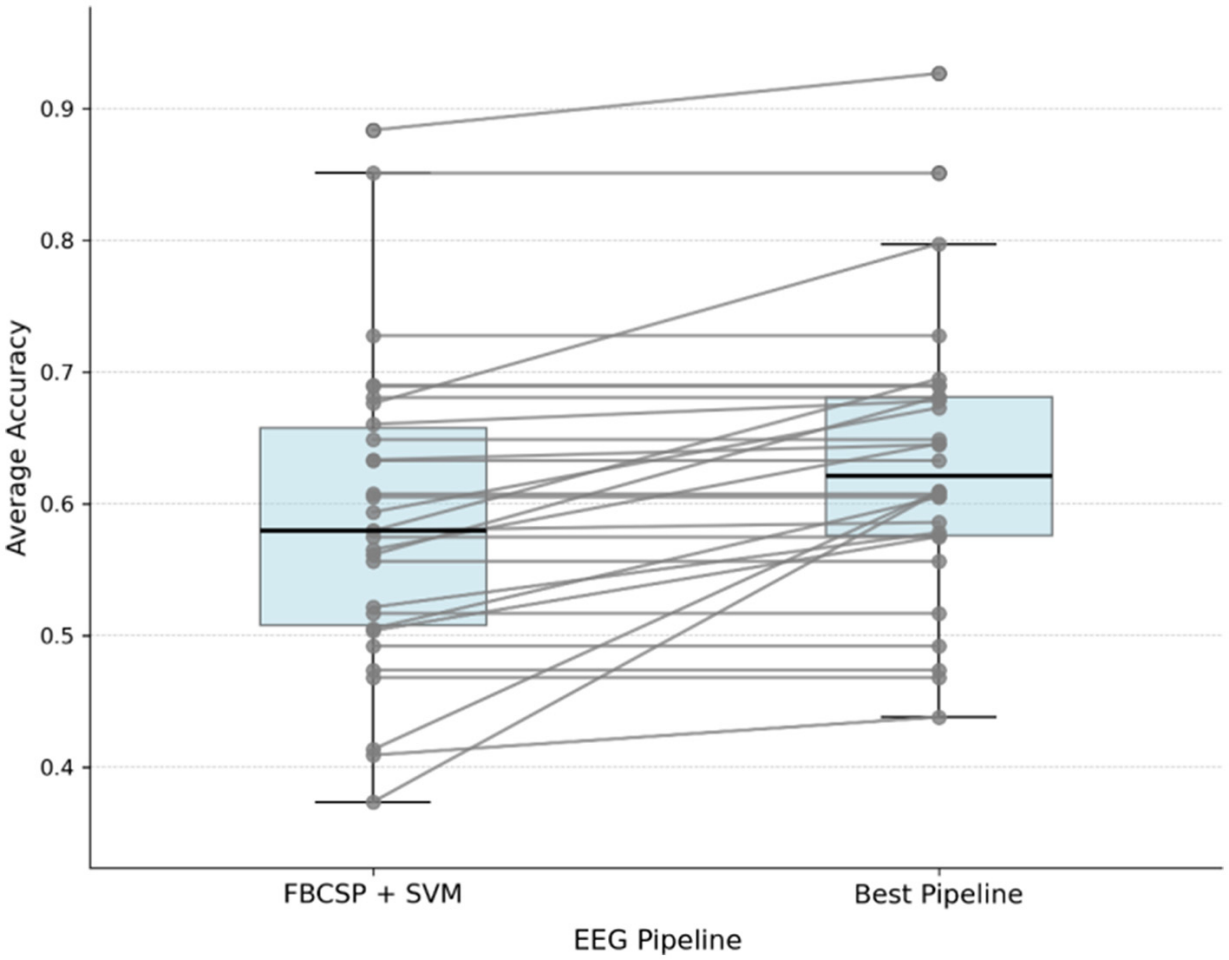
Post hoc analysis comparing offline decoder accuracy of our main EEG pipeline (FBCSP + SVM) vs. using the best pipeline per user (out of FBCSP + SVM, CSP + LDA, and Aug + Tang + SVM).

A *post hoc* analysis was performed to determine if offline accuracy could be improved using two alternative EEG decoding pipelines, CSP + LDA and Aug + Tang + SVM. After confirming the data was normally distributed, a one-way ANOVA showed no significant difference between the three tested pipelines [F_(2, 87)_ = 0.42, *p* = 0.66]. However, a paired *t*-test revealed that selecting the best-performing pipeline for each specific user resulted in significantly higher accuracy compared to using the FBCSP + SVM baseline for all users [*t* = –3.71, *p* < 0.001] (Figure 12). This highlights substantial individual variability in decoding performance, further confirmed by a significant participant effect ANOVA [F_(29, 60)_ = 9.53, *p* < 0.001].

## 6 Discussion

In this work, we introduced and evaluated a collaborative BRI system that combines eye tracking, EEG MI decoding, and EMG control for task execution in a kitchen environment. We compared three levels of autonomy (AT, SA, and FA) to examine how varying degrees of autonomy influence task performance, workload, ease of use, and agency.

Our results reveal a clear distinction between efficiency and reliability. In terms of user experience, participants clearly preferred the high-autonomy condition. FA (level 3) achieved the fastest task completion times, the lowest perceived workload (measured by NASA-TLX), and the highest system usability scores (measured by SUS), significantly outperforming the other conditions in these metrics. This confirms that for general usage, automating navigation and manipulation reduces the immediate physical and cognitive cost of interaction. Conversely, AT (level 1) resulted in the highest workload and lowest task success (13.3%), highlighting the prohibitive difficulty of manual gaze+EEG-based control for complex sequences.

However, while FA was preferred for its ease of use, SA (level 2) offered distinct advantages regarding system reliability. Statistically, both SA and FA significantly outperformed AT in task success. Yet, we observed a numerical drop in success for FA (66.7%) compared to SA (80.0%), highlighting a trade-off: FA relies on the noisy EEG MI decoder (59% accuracy) for decision-making, despite the workflow design being the least tolerant of classification errors. In contrast, SA limits the scale of potential errors by utilizing reliable eye-tracking to confirm object selection first, ensuring that any subsequent decoding errors are constrained within the context of a specific selected item.

Furthermore, SA may offer specific psychological benefits for the target population. While FA was rated easiest to use, trends in the SoPA scores (*p* = 0.065) suggest that users maintained a higher sense of agency in SA compared to FA. For healthy participants, the convenience of FA outweighed this loss of control. However, for individuals with severe motor impairments, this retention of agency is a critical factor for empowerment. Therefore, while FA is the optimal solution for efficiency, SA represents a valuable alternative for users who prioritize reliability and individuality.

It is also crucial to acknowledge that these results are inextricably linked to the specific interaction designs chosen for each level. For instance, our FA (level 3) condition relied exclusively on EEG for object selection, whereas SA leveraged the eye tracker. Had the FA (level 3) condition been designed to utilize eye tracking for selection, the performance gap between the levels may have shifted. Therefore, our findings should be interpreted as an evaluation of this specific multi-modal configuration, rather than a definitive claim that shared autonomy is superior to full automation across all potential interface designs.

Several limitations of this study should be acknowledged. First, our participant pool consisted entirely of healthy adults, which may not fully reflect the experiences, preferences, or performance of individuals with ALS or other severe motor impairments. Second, our participant sample exhibited a substantial gender imbalance, being heavily skewed toward male participants. Although prior work shows mixed and often context-dependent gender effects on usability, workload, and perceived agency—ranging from no measurable differences (Nuamah et al., 2019; Cachero et al., 2022) to task-specific modulations (Wienrich et al., 2024; Abbas and Jeong, 2024)—our unbalanced sample may still have influenced subjective ratings and limits the generalizability of our findings. Current evidence does not indicate consistent or systematic gender effects in HRI and BRI research (Winkle et al., 2023), but future studies should aim for a more gender-balanced sample and consider stratifying questionnaire responses by gender to more rigorously assess potential influences. Third, our sample size was relatively small, which may limit the generalizability of our findings. However, the statistical power of our analyses was largely preserved, as each participant completed all three autonomy levels. Fourth, EEG MI decoding achieved limited accuracy (0.59), which directly impacted the reliability and performance of FA. This emphasizes the importance of SA approaches that can compensate for current technical limitations while preserving user agency. Fifth, the system currently relies on a finite set of skills and robot policies, limiting flexibility, though integration with generalist robot policies (Ghosh et al., 2024; Black et al., 2024) could potentially expand automated object manipulation and task execution. Finally, real-world applicability still requires testing with assistive augmented reality interfaces, collision-aware navigation (for example, leveraging OSCBF), and user populations with motor impairments.

Another key outcome of this work is the demonstration that the extended M4Bench platform can support controlled experimentation on autonomy and agency in realistically complex assistive tasks. Beyond the levels of autonomy tested here, the same platform can be further extended to investigate other input modalities, biosignal-based decoders and paradigms, generalist robot manipulation policies, or even large foundation models for planning and control. While this current iteration is mainly oriented toward collaboration between users, the platform can be extended to other aspects of social interactions such as competition versus collaboration between users, task sharing, and delegation in the context of assistive systems.

In future works, additional tasks in the environment could increase engagement and ecological validity, while improved robot policies for navigation and manipulation could further enhance success rates. AT (level 1) could be made more granular, enabling precise robot control via EEG, and adaptive autonomy strategies could allow users to dynamically select their preferred assistance level. Real-world deployment could leverage commercially available devices, such as the Microsoft HoloLens mixed-reality headset with integrated EEG and eye tracking. Notably, Dillen et al. (2024) demonstrated the feasibility of integrating a BRI-based assistive system using this hardware, showing that practical translation of our system into everyday assistive contexts is possible. Furthermore, the performance of EEG offline decoding can vary widely between individuals (Figure 12), highlighting the potential benefit of customizing pipelines to each user to improve the usability of MI-based systems.

Overall, our findings demonstrate that while fully automated approaches are the most efficient and preferred by users, they remain brittle due to the reliance on noisy decoding within an error-intolerant workflow. In contrast, our shared autonomy level emerges as a critical design strategy, leveraging eye-tracking to constrain the impact of decoding errors to a verified context, ensuring reliable task execution without sacrificing the user's sense of agency.

## Data Availability

The datasets presented in this article are not readily available because of privacy concerns. Requests to access the datasets should be directed to the corresponding author.

## Footnotes

1 In contrast to the superset of *brain-machine interfaces* (BMIs) or *brain-computer interfaces* (BCIs), an important aspect of BRIs is to consider and develop robot control and behaviors, as well as real-time environmental feedback (Mao et al., 2017).

2 Executing direct end-effector control would be prohibitively difficult for our wide range of tasks.

3 https://github.com/vikashplus/furniture_sim/tree/master, https://free3d.com/, https://www.turbosquid.com/, and https://sketchfab.com/

4
https://pupil-labs.com/products/core

5
https://www.gtec.at/product/gnautilus-research/

## Appendix

**Table A1:** SUS questionnaire results (mean ± 1 standard deviation) split by autonomy level.

**Question**	**Autonomy level**		
	**AT**	**SA**	**FA**
I think that I would like to use this system frequently	1.5 ± 1.2	1.9 ± 1.3	2.7 ± 1.3
I did not find the system unnecessarily complex	2.2 ± 1.1	2.6 ± 1.2	3.1 ± 1.2
I thought the system was easy to use	1.5 ± 1.2	2.1 ± 1.1	2.7 ± 1.2
I do not think that I would need the support of a technical person to be able to use this system	1.3 ± 1.3	1.4 ± 1.4	2.5 ± 1.5
I found the various functions in this system were well integrated	2.3 ± 1.2	2.5 ± 1.2	3.0 ± 0.9
I did not think there was too much inconsistency in this system	2.3 ± 1.0	2.3 ± 1.3	3.1 ± 0.9
I would imagine that most people would learn to use this system very quickly	1.6 ± 1.3	2.1 ± 1.4	3.0 ± 1.1
I did not find the system very cumbersome to use	1.5 ± 1.0	2.1 ± 1.2	2.7 ± 1.4
I felt very confident using the system	1.5 ± 1.2	2.0 ± 1.4	2.7 ± 1.1
I did not need to learn a lot of things before I could get going with this system	1.7 ± 1.1	2.2 ± 1.3	3.0 ± 1.4

Responses are ranked from strongly disagree (1) to strongly agree (5). Usability improved with increasing automation, from the lowest scores in AT (level 1) to the highest scores in FA (level 3).

**Table A2:** SoARS questionnaire results (mean ± 1 standard deviation) split by autonomy level.

**Criterion**	**Autonomy Level**		
	**AT**	**SA**	**FA**
SoPA	4.82 ± 1.79	4.94 ± 1.70	4.16 ± 1.93
SoNA	3.23 ± 1.87	3.12 ± 1.75	3.33 ± 2.84

Participants reported higher SoPA under AT (level 1) and SA (level 2). There were small differences in the reported SoPA scores.
